# Antipsychotic pharmacogenomics in first episode psychosis: a role for glutamate genes

**DOI:** 10.1038/tp.2016.10

**Published:** 2016-02-23

**Authors:** J M Stevenson, J L Reilly, M S H Harris, S R Patel, P J Weiden, K M Prasad, J A Badner, V L Nimgaonkar, M S Keshavan, J A Sweeney, J R Bishop

**Affiliations:** 1 Department of Pharmacy and Therapeutics, University of Pittsburgh School of Pharmacy, Pittsburgh, PA, USA; 2 Department of Psychiatry and Behavioral Sciences, Northwestern University Feinberg School of Medicine, Chicago, IL, USA; 3 Jesse Brown Veterans Administration Medical Center, Chicago, IL, USA; 4 Department of Pharmacy Practice, College of Pharmacy, University of Illinois at Chicago, Chicago, IL, USA; 5 Department of Psychiatry, University of Illinois at Chicago, Chicago, IL, USA; 6 Department of Psychiatry, Western Psychiatric Institute and Clinic, Pittsburgh, PA, USA; 7 Department of Psychiatry, University of Chicago, Chicago, IL, USA; 8 Department of Human Genetics, University of Pittsburgh, Pittsburgh, PA, USA; 9 Department of Psychiatry, Harvard Medical School, Boston, MA, USA; 10 Department of Psychiatry, University of Texas Southwestern Medical Center, Dallas, TX, USA; 11 Department of Experimental and Clinical Pharmacology, University of Minnesota, Minneapolis, MN, USA

## Abstract

Genetic factors may underlie beneficial and adverse responses to antipsychotic treatment. These relationships may be easier to identify among patients early in the course of disease who have limited exposure to antipsychotic drugs. We examined 86 first episode patients (schizophrenia, psychotic bipolar disorder and major depressive disorder with psychotic features) who had minimal to no prior antipsychotic exposure in a 6-week pharmacogenomic study of antipsychotic treatment response. Response was measured by change in Brief Psychiatric Rating Scale total score. Risperidone monotherapy was the primary antipsychotic treatment. Pharmacogenomic association studies were completed to (1) examine candidate single-nucleotide polymorphisms (SNPs) in genes known to be involved with glutamate signaling, and (2) conduct an exploratory genome-wide association study of symptom response to identify potential novel associations for future investigation. Two SNPs in *GRM7* (rs2069062 and rs2014195) were significantly associated with antipsychotic response in candidate gene analysis, as were two SNPs in the human glutamate receptor delta 2 (*GRID2)* gene (rs9307122 and rs1875705) in genome-wide association analysis. Further examination of these findings with those from a separate risperidone-treated study sample demonstrated that top SNPs in both studies were overrepresented in glutamate genes and that there were similarities in neurodevelopmental gene categories associated with drug response from both study samples. These associations indicate a role for gene variants related to glutamate signaling and antipsychotic response with more broad association patterns indicating the potential importance of genes involved in neuronal development.

## Introduction

It is well-established that treating psychotic illness with antipsychotic medications improves outcomes and reduces relapse rates,^1^ but treatment response and tolerability are highly variable and many patients have unremitting symptoms.^2^ Thus, there is a clinical need to reliably identify patients who may require different or adjunctive treatment strategies, and to understand biological mechanisms of treatment response.^3^

Aberrant glutamate transmission is hypothesized to be a significant contributor to symptoms and cognitive impairment in psychotic disorders.^4^ Drugs that antagonize *N*-methyl-d-aspartic acid (NMDA) receptors in patients and in animal models have effects which mimic the positive, negative and cognitive symptoms of psychosis.^5^ Consistent with this, glutamate system genes including *GRIA1*, *GRIN2A* and *GRM3* have been associated with disease risk in recent analyses from the Psychiatric Genomics Consortium.^6^ Glutamate gene polymorphisms are also associated with neurocognitive endophenotypes in genetic studies of schizophrenia.^7, 8^

Some evidence suggests that aberrant glutamatergic function may alter dopamine system function in psychotic disorders.^9, 10, 11^ For example, NMDA antagonism potentiates the dopaminergic response to an amphetamine challenge.^12^ Furthermore, dopamine D2/D3 receptor availability is linked to the severity of psychotic symptoms induced by NMDA antagonism.^13^ Thus, factors influencing glutamate signaling may contribute to dopamine dysregulation and variability in symptoms or response to dopamine-mediated treatments.

Psychosis-related behaviors and symptoms induced by NMDA receptor antagonism can be reversed by agents that influence either glutamate or dopamine receptor activity.^14, 15, 16, 17^ Although all currently available antipsychotic agents share dopamine receptor antagonism as a common mechanism of action, single-nucleotide polymorphisms (SNPs) in glutamate-related genes have been associated with antipsychotic response or treatment resistance.^18, 19, 20, 21^ These studies examined candidate polymorphisms in the type-3 metabotropic glutamate receptor gene (*GRM3*), but the roles of other genes that encode glutamate receptors or transporters have not yet been as systematically investigated.

In the present study, we examined pharmacogenomic relationships between glutamate gene polymorphisms and antipsychotic response in first episode psychosis patients who had received minimal prior antipsychotic exposure. Treatment with antipsychotics is a common pharmacotherapy approach for psychosis in the context of schizophrenia, psychotic bipolar disorder or major depressive disorder with psychosis. In addition, glutamatergic mechanisms may be related to each.^22^ Given the overlaps in symptom presentation and standard treatment, there may also be similar genetic characteristics underlying variability in treatment response. Studies of pharmacogenetic influences in first episode patients have the important advantage of very limited exposure to prior antipsychotic drug treatment, which can itself alter receptor expression,^23^ brain function^24, 25^ and brain structure.^25, 26, 27^ Thus these studies may provide clearer insights into genetic associations with treatment response. In addition to candidate gene studies, we conducted a hypothesis-generating genome-wide association analysis to identify avenues for potential future research. This to our knowledge represents the first genome-wide association analysis of antipsychotic response in first episode psychosis.

## Materials and methods

### Ethics statement

This study was approved by the Institutional Review Boards at the University of Illinois at Chicago and the University of Pittsburgh where subjects were recruited. Patients provided written informed consent.

### Study population

Eighty-six patients meeting Diagnostic and Statistical Manual of Mental Disorders (DSM-IV) criteria for schizophrenia, schizoaffective disorder, psychotic bipolar disorder or major depressive disorder with psychotic features were recruited. Diagnoses were determined using the Structured Clinical Interview for DSM Disorders (SCID)^28^ at consensus diagnostic meetings. Participants were outpatients or inpatients at the University of Illinois at Chicago (*n*=40) or the University of Pittsburgh Western Psychiatric Institute and Clinic (*n*=46). Participants were either antipsychotic naive (*n*=68) or had received minimal prior antipsychotic exposure (*n*=18) defined as cumulative lifetime exposure <18 weeks (median reported lifetime exposure of 21.5 days). Those with prior antipsychotic exposure were untreated during baseline study assessments. Patients had not received treatment with anticonvulsants or lithium prior to enrollment. Participants receiving antidepressants at enrollment were included if therapy was determined to not be therapeutically beneficial to psychosis symptoms. Participants were at least 15 years of age, with no history of head trauma and no active substance or alcohol abuse or lifetime history of substance dependence. We conducted our primary analyses on the whole study sample of those with psychosis and repeated analyses in the subgroup of patients with a schizophrenia-spectrum diagnosis (schizophrenia or schizoaffective disorder—depressed subtype) as well as the subgroup of patients treated with risperidone.

### Treatment protocol and outcomes

Consenting individuals were treated with 6 weeks of open-label, flexibly dosed antipsychotics. Risperidone monotherapy was the antipsychotic therapy of choice, but prescribers could opt for alternative antipsychotic agents where clinically preferred. Chlorpromazine (CPZ) equivalents were calculated to allow for examination of antipsychotic dose across patients treated with different antipsychotic agents.^29^ Symptoms were evaluated using the Brief Psychiatric Rating Scale (BPRS) total score^30^ administered by senior clinician evaluators, each with extensive experience using this scale and with the patient population. Independent group ratings of patients were performed on a periodic basis to ensure consistency symptom evaluation over time. Change score on the BPRS from pre to post treatment was the primary clinical outcome, calculated so that higher positive numbers represent better clinical improvement. Changes on the BPRS positive and negative subscales were evaluated as secondary outcomes.

### Genotyping and DNA microarray quality control

Genomic DNA was isolated from EDTA-treated whole blood using the Gentra Puregene extraction kit (Qiagen Sciences, Germantown, MD, USA), quantified and quality checked with Picogreen (Invitrogen, Eugene, OR, USA) and Nanodrop assays (Thermo Scientific, Wilmington, DE, USA).

Array genotyping was performed at the Translational Genomics Research Institute using standard protocols for the Affymetrix Genome-Wide Human SNP Array 6.0 (Affymetrix, Santa Clara, CA, USA).^31^ Follow-up and confirmatory genotyping were performed in our laboratory using sequence validated assays. Genetic analyses were performed after study completion of the study and thus assessments were blinded to genotyping results. A minimum call rate of 95% was required for each sample to be included in analyses; all samples met this criterion. We confined primary analyses to SNPs with minor allele frequencies (MAF) ⩾10% to focus on more common variation given our sample size. All genetically inferred sex determinations matched those obtained by self-report. Identity-by-descent analyses were used to identify and exclude duplicated or contaminated samples; no samples were excluded for this reason. The final data set included 86 individuals and 600 577 autosomal SNPs that met the following criteria: (1) Genotype call rate of ⩾95% (2) MAF ⩾10% (3) Hardy–Weinberg Equilibrium *P*>1 × 10^−6^. Genotypes for significantly associated SNPs on the array were confirmed with TaqMan assays (Thermo Fisher, Waltham, MA, USA).^21^ Genetic regions exceeding genome-wide significance in our analysis of common (MAF ⩾10%) SNPs were further examined by interrogating other less common SNPs with MAF <10% (for a total of 750 347 SNPs) as well as targeted genotyping of amino-acid-altering variants (rs10034345 and rs34144324) and an insertion/deletion polymorphism (rs10681348) not represented on the array. TaqMan assays were used for rs10034345 and rs34144324, genotyping for rs10681348 was performed by Sanger sequencing (see Supplementary Methods).

### Demographics and genetic association models

Stata (StataCorp, College Station, TX, USA) was used to evaluate clinical and demographic characteristics and for *post hoc* modeling of individual SNPs. Clinical and demographic differences across diagnoses were evaluated using one-way analysis of variance (ANOVA) or *χ*^2^ statistics. Paired *t*-tests were used for pre–post-treatment changes in BPRS scores. Genetic association tests examined change in BPRS score as a quantitative phenotype. This method modeled BPRS change score using a linear regression with SNPs assessed using an additive genetic model. All analyses were adjusted for baseline BPRS score, CPZ equivalent dose, diagnosis and ancestry. For secondary analyses of BPRS positive and negative subscales, the baseline BPRS score covariate corresponded to the secondary phenotype (for example, baseline BPRS positive subscale for the analysis of BPRS positive change score). Study site was not a significant term in our regression model (*P*=0.81). CPZ equivalent dose was natural log-transformed to improve normality. *Post hoc* adjusted mean change in BPRS score by genotype was calculated using these linear regression models.

We used principal component analyses to control for population admixture in our study sample where *k*=3 primary principal components were identified and thus *k*−1=2 components were included in regression models, which resulted in a lack of substantial genomic inflation (Supplementary Figure 1). As additional checks to identify the potential influence of ancestry on findings of interests, we examined significant associations from the primary genome-wide analysis in strata defined by self-identified race. We also repeated main analyses using a specific group of 105 ancestry informative markers^32^ as well as self-identified race indicator variables in place of principal components. These along with race-group stratified analyses of our primary finding confirmed no significant effect of race/ancestry on study findings.

### Glutamate candidate gene analyses

PLINK v1.07^33^ was used to examine candidate gene associations. Forty-nine genes encoding glutamate receptors, downstream signaling components and enzymes involved in the transport, secretion and metabolism of glutamate were included (Table 1).^34, 35^ SNPs meeting quality control parameters within these genes ±50 kb were included in analysis.

We first used the set-based test feature in PLINK with 100 000 permutations to identify up to 5 SNPs in each gene that were independent of one another (*r*^2^<0.5) and nominally significant (*P*<0.05). Our second step was to examine the association between these nominally significant SNPs and response using 100 000 permutations to generate experiment-wise *P*-values, which represent how often permuted data sets created an association at least as strong as that which was originally observed. SNPs with experiment-wise *P*-values <0.05 were considered statistically significant.

### Genome-wide analyses

Golden Helix SNP & Variation Suite version 8.3.0 (Golden Helix, Bozeman, MT, USA) was used to analyze genome-wide data. Genome-wide significance threshold was set at the standard *P*-value threshold of 5 × 10^−^^8^.^36^ Given that our final analyses included 750 347 SNPs, this is slightly more conservative than a Bonferroni adjustment at *α*=0.05 (*P*=6.6 × 10^−8^).

### Examination of findings in a separate risperidone study sample

We further examined consistencies and overlaps of our findings with published data from risperidone-treated participants (*n*=240) of the Clinical Antipsychotic Trials of Intervention Effectiveness (CATIE) schizophrenia study.^37^ Details on our approach are included in the Supplementary Methods. Briefly, we examined our findings in two ways. The first was a direct examination of our primary SNP findings from both candidate gene and GWAS studies reported herein. Second, we examined the top 500 SNP associations from both our study and the CATIE risperidone study sample with Ingenuity Pathway Analysis (IPA, Redwood City, CA, USA http://www.qiagen.com/ingenuity). In this analysis we: (a) tested the hypothesis that top SNPs were overrepresented in glutamate system genes relative to expectation, as well as (b) used a hypothesis-free discovery analysis to more broadly determine whether there were commonalities in the pathways or categories of genes most highly associated with treatment response in the two studies.

### Correlation of identified variants with gene expression and imaging phenotypes

To further assess the biological plausibility of our findings, we examined our primary SNP associations in publicly available databases for SNP by gene expression relationships in the brain. Genomic imaging resources included dorsolateral prefrontal cortex expression data via BrainCloud (http://braincloud.jhmi.edu/),^38^ and other brain regions via the UK Brain Expression Consortium (BRAINEAC; http://www.braineac.org/).^39^

## Results

### Symptom improvement

Pre-treatment characteristics of the patient sample are summarized in Table 2. Symptoms significantly improved after treatment (*P*<0.0001) with a mean BPRS score improvement (±s.d.) of 9.1±0.8. There were differences in pre-treatment BPRS scores across diagnoses (*P*<0.05) but the primary outcome, BPRS change score, did not significantly differ across diagnoses (*P*=0.59).

### Glutamate candidate gene panel association study

Set-based analyses identified a total of 87 linkage disequilibrium-independent nominally significant SNPs in 30 genes. Experiment-wise *P*-values for two SNPs in *GRM7*, rs2069062 and rs1532544, were statistically significant (*P*<0.04). The 10 strongest associations from the candidate gene study are displayed in Supplementary Table 1. Adjusted mean change scores (±s.d.) on the BPRS for the most robust finding, *GRM7* rs2069062, were CC 10.4±4.7, CG 6.7±4.8 and GG 5.4±3.9 (*P*<0.05). In the subset of individuals with schizophrenia-spectrum illness, we identified 92 linkage disequilibrium-independent nominally significant SNPs in 31 genes. Experiment-wise *P*-values for rs2069062 in *GRM7* and rs598134 in *GRM5* were statistically significant (Supplementary Table 2). Genomic imaging database analyses identified significant associations between both of these variants and the expression of their respective genes in the substantia nigra (rs2069062 *P*=0.008 and rs598134 *P*=0.00016) as well as the white matter for *GRM7* (rs2069062 *P*=0.0018).^41^

### Exploratory genome-wide association study

**Figure 1:**
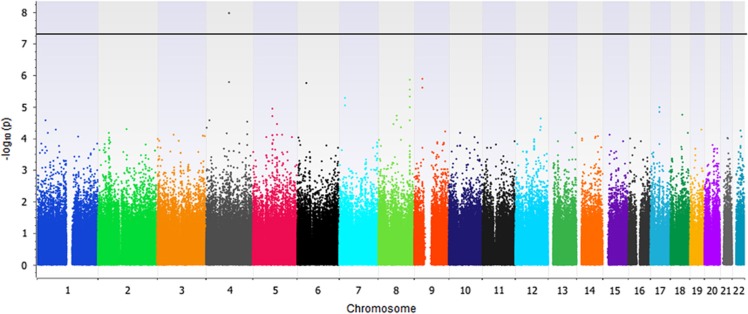
Manhattan plot of BPRS change scores after 6 weeks of antipsychotic treatment. BPRS, Brief Psychiatric Rating Scale.

**Figure 2:**
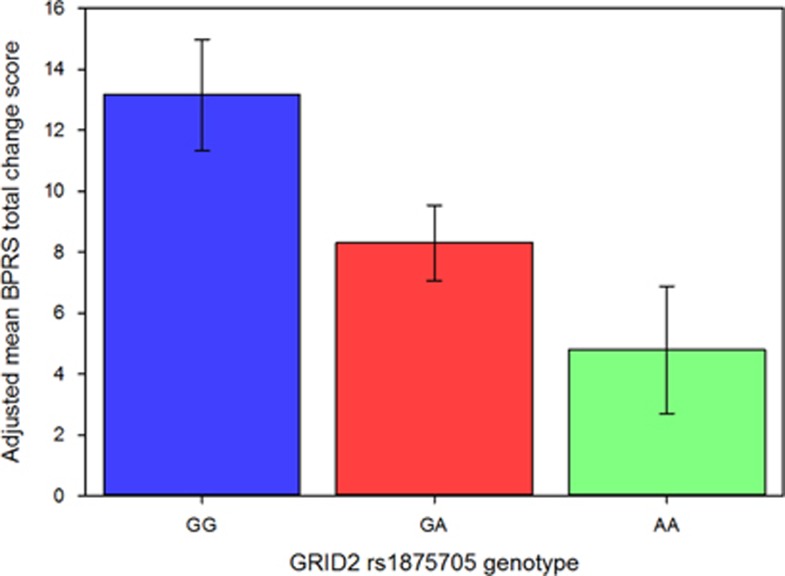
BPRS change scores by *GRID2* rs1875705 genotype (95% CI). BPRS, Brief Psychiatric Rating Scale; CI, confidence interval.

Genome-wide association tests identified significant relationships between two glutamate receptor delta 2 (*GRID2*) variants and BPRS change score (*P*=1.10 × 10^−8^, Figure 1 and Table 3). These SNPs, rs9307122 and rs1875705, are located 10 kb from one another and were in complete linkage disequilibrium in the study sample. Neither SNP was significantly associated with change on positive nor negative symptom subscales in secondary analyses of schizophrenia-only subjects (Supplementary Tables 3 and 4). Publicly available genomic imaging data identifies both of these SNPs as significantly associated with *GRID2* expression in the hippocampus (*P*=0.0087 and *P*=0.0089, respectively).^41^ Differences in BPRS change across genotype groups presented in an additive manner (Figure 2). The next-nearest *GRID2* SNP on the array, rs994011, approached, but did not meet the genome-wide significance threshold (*P*=1.64 × 10^−6^). Associations were consistent across race groups (Supplementary Table 5). Primary findings were unchanged when repeating analyses using ancestry informative markers or self-identified race indicator variables in place of principal components. Variables for ancestry were not significant in models of BPRS change score (*P*>0.05). Supplementary Figure 1 is a Q–Q plot from the main genome-wide analysis, and the Supplementary Table 8 includes the 500 SNPs of strongest association from genome-wide analysis.

Two additional variants in *GRID2*-coding regions (rs34144324 Thr68Met and rs10034345 Val395Ile) located <1 mb from our associated variants, but not included on the array, were identified and subsequently genotyped. Both SNPs represent coding non-synonymous polymorphisms. The SNP rs10034345 was nominally associated with change in BPRS score (*P*=0.05) and rs34144324 was not associated (*P*>0.05). An insertion/deletion polymorphism (rs10681348) located between the significant SNPs identified in genome-wide analyses was also genotyped and showed a nominal association with change in BPRS score (*P*=0.01).

We performed *post hoc* analyses to examine whether findings remained significant in subgroups of diagnostic and treatment homogeneity. In the subgroup of patients with schizophrenia-spectrum illness (*n*=68), the same two *GRID2* SNPs from the primary analysis (rs9307122 and rs1875705) were significantly associated with change score on the BPRS (*P*=2.3 × 10^−8^, Supplementary Table 6, Supplementary Figures 2 and 3). In addition, another SNP (rs687279) in an intergenic region of chromosome 8 reached genome-wide significance in this secondary analysis (*P*=1.4 × 10^−8^). The association of *GRID2* rs9307122 and rs1875705 with BPRS total scores remained robust (*P*=5.4 × 10^−8^) in the subgroup of patients receiving risperidone (*n*=68).

### Examination of findings in the CATIE risperidone treatment group

In the CATIE risperidone treatment group, there were *n*=10 SNPs identified in *GRM7* that had nominally significant (*P*=0.00054–0.024) associations with PANSS total change scores as previously reported.^37^ All but one of these markers was genotyped in our study and not significantly associated with response in our analyses. Our primary *GRM7* finding (rs2069062) was not directly associated with symptom improvement in CATIE although other *GRM7* SNPs (rs7627369 and rs6774660), located ~600 kb from our top finding were nominally associated with symptom response in previous candidate gene studies of CATIE.^42^

*GRID2* SNPs identified from our GWAS analysis as well as secondary targeted SNP assays were not included in the DNA microarrays used to genotype participants in the CATIE study. There were *n*=14 *GRID2* SNPs identified that had nominal associations (*P*=0.011–0.041) with PANSS total change scores in that study sample. Of this SNP set, rs12506519 was nominally associated with PANSS total and BPRS total improvement scores for the CATIE and first episode study samples, respectively (*P*=0.04 in both study samples).

A more broad examination of the top 500 SNPs associated with response in our first episode and the CATIE risperidone study samples was completed with IPA. These top 500 variants represented associations with symptom change scores during treatment at the *P*=0.0016 and *P*=0.0087 levels of significance or better for the CATIE and first episode study samples, respectively. These top SNPs were overrepresented in genes related to glutamate signaling (first episode *P*=0.000184 and CATIE *P*=0.00000393). A hypothesis-free pathway analysis identified that the top Physiological System Development and Function category represented in both study samples was the broad classification of Nervous System Development and Function. Within this classification, the top two functional annotations identified for both study samples were also the same. Neuritogenesis (FE *P*=2.03E−05, CATIE *P*=7.0E−09) and neuronal development (FE *P*=8.42E−05, CATIE *P*=5.91E−05) were significantly overrepresented relative to expectation in each study sample. Interestingly, different genes in each study sample represented the findings for these functional annotations (See Supplementary Table 7).

## Discussion

We conducted both candidate gene and genomic analyses to examine pharmacogenomic relationships with antipsychotic response in first episode psychosis. Candidate gene analyses were notable for variations of potential interest in *GRM7,* which has been investigated in studies of schizophrenia, ADHD and autism.^43, 44, 45^ In exploratory genome-wide analyses, the findings of greatest statistical significance were in *GRID2*, a gene with evidence suggesting involvement in glutamate signaling. Further examination of our findings in risperidone-treated subjects from the CATIE study revealed that top SNPs in both studies were included in glutamate system genes more than expected by chance and further that there were consistencies in neuron development gene categories represented by the SNPs most highly associated with response in each study. These findings collectively lend support for the hypothesis that genetic variation in glutamate system genes may impact the clinical trajectory of first episode patients treated with antipsychotic medications, and that these may represent a broader involvement of neurodevelopmental pathways.

### Candidate gene study findings

Findings from our candidate gene analyses identified significant symptom response associations with SNPs in *GRM7*. The metabotropic glutamate receptor 7 encoded by *GRM7* maps to chromosome 3p26.1–p25.2.^46^ It encodes the G-protein coupled mGluR7 receptor, which is a member of the type-III group of metabotropic glutamate receptors and is expressed in cerebellum, neocortex and hippocampus.^47, 48^ The mGluR7 receptors are located at presynaptic terminals of glutamatergic and GABAergic neurons. They inhibit the release of glutamate or GABA when activated^48^ and regulate NMDA receptor trafficking and function.^49, 50^ *GRM7* knockout mice exhibit epilepsy as well as diverse behavioral problems.^51, 52, 53, 54^

NMDA receptor dysregulation has been strongly associated with the pathophysiology of schizophrenia,^55^ with imaging studies suggesting glutamate disposition related to treatment resistance,^56^ and the type-III mGluRs such as mGluR7 appear most functional in situations of higher glutamate transmission, serving as a ‘brake' on glutamate release when bound.^50^ The *GRM7* SNP most strongly associated with symptom response in our analysis was rs2069062 and also noted to be associated with whole brain white matter in previous genomic imaging studies.^39, 41^ When we specifically examined associations from risperidone-treated subjects in CATIE there was evidence for associations in that gene in both study samples, although there was not direct overlap of the specific SNPs.

### Genome-wide association findings

The most striking findings from genome-wide analyses were associations in the ionotropic *GRID2* gene, which encodes the GluD2 receptor protein. These associations exceeded genome-wide significance in both the complete sample and in an analysis restricted to patients with a schizophrenia-spectrum diagnosis and were also robust in analyses restricted to patients receiving risperidone. In a publicly searchable brain expression quantitative trait loci database of healthy brains,^41^ variation in our most significant *GRID2* SNPs were associated with hippocampal GluD2 mRNA expression. Specifically, the rs1875705 A/A genotype, which was associated with reduced response in our study, was associated with lower hippocampal expression. We also identified and genotyped two nearby exonic missense mutations (rs10034345 and rs34144324) which were not captured on the array, as well as an in-frame insertion/deletion. Associations for rs10034345 and rs10681348 were statistically significant (*P*<0.05), but neither of these polymorphisms showed stronger associations than the originally identified SNPs (rs9307122 and rs1875705). While these variants may not be the causal polymorphisms for the association, haplotype effects cannot be ruled out and require further investigation. The SNPs most highly associated with treatment response in our first episode population were not directly assayed in the CATIE study. However, we identified regions of *GRID2* nominally associated with response in both study samples that provide a basis for further examination of the importance of this gene.

*GRID2* maps to chromosome 4q22.1-22.2^57^ and GluD2 knockouts are notable for motor, learning and cognitive deficits in animal models.^58, 59, 60, 61^ *In vitro* experiments demonstrate interactions between GluD2 and AMPA (via the GluR1 subunit) as well as kainate receptors (via the GluR6 subunit).^62^ Additional expression studies indicated that co-expressing wild-type GluD2+GluR1 resulted in a reduction of glutamate-induced signaling currents through AMPA receptors, an effect that was not observed when altered/dysfunctional GluD2 was co-expressed with GluR1 in similar experiments.^62^ These findings along with previous studies indicate that altering GluD2 disrupts AMPA receptor signaling.^63, 64^

Antipsychotics, notably risperidone, increase the expression or affinity of AMPA receptors.^65, 66^ This may be due to an indirect mechanism such as compensatory upregulation due to antipsychotic antagonism at 5HT2A or D2 receptors, or through post-translational mechanisms. It has been proposed that this mechanism contributes to the clinical benefit of antipsychotic treatment by normalizing glutamate hypofunction in schizophrenia.^65, 66^ Given what we know about the importance of functional GluD2 for APMA activity, our findings provide an important basis for future studies investigating GluD2 and the mechanism of treatment response. Whether dysfunctional GluD2 or other cofactors influencing AMPA signaling are differentially compensated for by antipsychotic treatment is a novel avenue for mechanistic research into sources of variability in antipsychotic response and may potentially provide a novel strategy for drug development.

Limited data from other studies provide support for the potential importance of *GRID2* for familial phenotypes associated with psychotic disorders, as well as in response to drugs that act through glutamatergic mechanisms. *GRID2* is noted to be associated with neurocognitive endophenotypes including prepulse inhibition, startle habituation, P50 suppression, verbal learning and executive function tasks.^7, 8^ In addition, our most significant SNP, rs1875705, was nominally associated with schizophrenia is the Psychiatric Genomics Consortium's mega-analysis.^6^ New evidence linking *GRID2* deletion with altered response to the NMDA receptor antagonist memantine further supports the importance of intact GluD2 function for optimal glutamate signaling.^67^

Whether the associations seen in previous research represent interactions with treatment status is unknown, as those findings were observed in chronically treated patients. Presently, no other pharmacogenomic studies have examined *GRID2* in the context of antipsychotic treatment for early episode psychosis or schizophrenia.

### Potential importance of genes involved in neurodevelopment

Through our efforts to examine consistencies with another risperidone treatment study sample, we identified interesting findings of potential importance. The first is that despite the notable differences in study design and chronicity/treatment histories of patients involved, genes involved with neuronal development were robustly overrepresented relative to expectation when we broadly examined top tier findings from both studies. Furthermore, the genes associating with this categorization were largely different for each group. While we did not embark on this effort as an exhaustive bioinformatics exercise, it is worth noting the potential significance and implications of this observation. Although our data further support the importance of glutamate system genes in treatment response, their involvement may also be part of a broader picture and that perhaps variation in neuronal development somehow alters brain structure or function in ways that translate to drug sensitivity. Perhaps equally, if not more important, is that replication of gene groups or pathways is an important approach to complement direct SNP or gene replication efforts.

Our study must be interpreted in the context of its limitations. While samples of untreated first episode psychosis patients can potentially be very informative, because of their ability to examine treatment effects unconfounded by effects of prior treatment they are by nature small in size. Thus, statistical power may not have been sufficient to identify associations with smaller effect and/or with rare variants. Significant associations from our genome-wide studies should ideally be replicated in independent data sets.^36^ In this effort, we found some consistencies across study samples, although there were notable differences in studies that preclude us from categorizing this as a true replication effort. Using publicly available imaging genomics data provides some information that our findings may be relevant to brain function. In addition, our sample included individuals with psychotic symptoms but with differing clinical diagnoses. We recognize that there may be differences in drug response across diagnoses, but we did consider this in our analytical approach. Furthermore, our sample consisted of individuals with differing race and ethnicity, which we addressed in analyses but also requires careful assessment of findings. We examined the influence of race in numerous ways which did not alter our primary findings, but effects of ancestry cannot be fully discounted. Finally, a few subjects received antipsychotic agents other than risperidone. We adjusted analyses for equivalent antipsychotic dose, which is a method that is helpful in adjusting for dosing across different antipsychotic agents, but potential pharmacogenetic differences across specific antipsychotic medications are a potential consideration. Notably our top findings remained robustly associated with response phenotypes in analyses restricted to risperidone-treated patients, perhaps reducing this concern.

In conclusion, we report pharmacogenomic associations with antipsychotic response that indicate an important role of glutamate system genes and perhaps more broadly neuronal development in the treatment trajectory of patients with psychotic illness. Notably, we identified a novel but biologically plausible relationship between *GRID2* and antipsychotic response. In our candidate gene analyses, we identified potential regions-of-interest in *GRM7*. These findings represent exciting and novel associations for further investigation in antipsychotic pharmacogenomic studies.

## Figures and Tables

**Table 1:** Glutamate-related candidate genes

*Ionotropic receptors*		*Metabotropic receptors*	*Downstream signaling*		*Transport and secretion*		*Metabolism*
*GRIK1*	*GRIA4*	*GRM1*	*RGS4*	*GNAQ*	*ADORA1*	*SLC17A8*	*SNCA*
*GRIK2*	*GRIN1*	*GRM3*	*ADCY7*	*HOMER1*	*IL1B*	*SLC1A1*	*ALDH5A1*
*GRIK3*	*GRIN2A*	*GRM4*	*APP*	*HOMER2*	*P2RX7*	*SLC1A2*	*GAD1*
*GRIK5*	*GRIN2B*	*GRM5*	*CACNA1A*	*ITPR1*	*SLC17A6*	*SLC1A3*	*GLS*
*GRIA1*	*GRIN2C*	*GRM7*	*CLN3*	*MAPK1*		*SLC1A6*	*GLUL*
*GRIA2*		*GRM8*	*DLG4*	*PLA2G6*		*SLC38A1*	*PRODH*
			*GNAI1*	*PLCB1*		*SLC7A11*	
				*SHANK2*			

**Table 2:** Sample characteristics by diagnosis

	*Full sample (*n=*86)*	*Sz (*n=*68)*	*BPD (*n=*11)*	*MDD (*n=*7)*
*Pre-treatment characteristics*				
Age±s.d.	24.2±6.9	24.1±6.4	26.7±9.6	21.6±6.9
Male (%)	55 (64)	47 (69)	5 (45)	3 (43)
* Self-identified race (%)*				
White	37 (43)	32 (47)	2 (18)	3 (43)
Black	35 (41)	27 (40)	6 (55)	2 (29)
Other	14 (16)	9 (13)	3 (27)	2 (29)
Antipsychotic naive (%)	68 (79)	55 (81)	8 (73)	5 (71)
Pre-treatment BPRS^30^ score±s.d.*	45.3±9.2	46.8±9.0	37.3±7.9	42.7±6.7
				
*Post-treatment characteristics*				
* Treatment agent*				
Risperidone monotherapy (%)	67 (78)	50 (74)	11 (100)	6 (86)
Other SGA monotherapy (%)	8 (9)	7 (10)	0 (0)	1 (14)
FGA monotherapy (%)	10 (12)	10 (15)	0 (0)	0 (0)
SGA+FGA (%)	1 (1)	1 (1)	0 (0)	0 (0)
CPZ equivalents±s.d.^29,40^	249.2±172.9	271.0±181.2	180.5±83.5	145.0±136.8
Taking benztropine (%)	15 (17)	15 (22)	0 (0)	0 (0)
Taking antidepressant (%)	14 (16)	9 (13)	0 (0)	5 (45)
Taking mood stabilizer (%)	2 (2)	1 (1)	1 (9)	0 (0)
Taking sedative/hypnotic (%)	3 (3)	3 (4)	0 (0)	0 (0)
BPRS post-treatment score±s.d.	36.1±9.0	38.1±8.6	27.1±4.6	31.1±8.0
BPRS change score±s.d.	9.1±7.8	8.7±7.7	10.2±9.2	11.6±6.3

Abbreviations: BPD, bipolar disorder; BPRS, Brief Psychiatric Rating Scale; BPRS change score, pre-treatment score minus post-treatment score; CPZ equivalents, chlorpromazine equivalents; FGA, first generation antipsychotic; MDD, major depressive disorder; SGA, second generation antipsychotic; Sz, schizophrenia.

*P<0.05 between diagnoses.

**Table 3:** Twenty strongest associations from genome-wide analysis

*Rank*	*Chromosome*	*rsID*	*Gene*	*Region*	*−log*_*10*_ P*-value*
1	4	rs9307122	*GRID2*	Intron	7.96
2	4	rs1875705	*GRID2*	Intron	7.96
3	9	rs3824457	*APTX*	Intron	5.88
4	8	rs1992525	Intergenic	—	5.85
5	4	rs994011	*GRID2*	Intron	5.79
6	6	rs804855	*MDGA1*	Downstream	5.75
7	9	rs2274766	*SMU1*	Intron	5.60
8	8	rs687279	Intergenic	—	5.54
9	8	rs2288687	Intergenic	—	5.32
10	7	rs16100	*NPY*	Downstream	5.29
11	7	rs16101	*NPY*	Downstream	5.05
12	8	rs6981424	Intergenic	—	4.98
13	17	rs2254914	*ACACA*	Intron	4.98
14	5	rs256447	*THBS4*	Intron	4.94
15	17	rs2542660	*ACACA*	Intron	4.84
16	18	rs12954691	*MRO*	Upstream	4.74
17	8	rs1971364	*CRISPLD1*	Downstream	4.72
18	5	rs256444	*THBS4*	Intron	4.71
19	12	rs3817602	*GLT8D2*	Exon	4.62
20	8	rs1455796	*CRISPLD1*	Intron	4.59
