# Supplementary material for: Antipsychotic pharmacogenomics in first episode psychosis: a role for glutamate genes
Source: Transl Psychiatry. 2016 Feb 23;6(2):e739–. doi: 10.1038/tp.2016.10 (PMC4872428; doi:10.1038/tp.2016.10)
Supplement: Supplementary Information [file tp201610x1.pdf]

Supplementary Methods

Targeted genotyping

Targeted genotyping was done to validate microarray findings of interest as well as to examine other potential variants of interest not covered on the array. TaqMan assays (C_25932070_20 and C_25998863_10) were used for rs10034345 and rs34144324. Genotyping for rs10681348 was performed by PCR and Sanger sequencing, with F primer GAGCACTGGGTTTCTCTCTCA and R primer TGCCTAAACAATGCTAAATGGA . Each PCR consisted of 25 µl HotStarTaq Mastermix, 25 pmol of each primer and 20-100 ng of DNA. The amplification cycle consisted of an initial denaturation at 95° C for 15 min, 30 cycles of denaturation at 95° C for 30 sec, annealing at 53° C for 30 sec, extension at 72° C for 45 sec, and a final extension at 72° C for 10 min. PCR products were purified prior to Sanger sequencing using the Wizard SV 96 PCR Clean-Up System (Promega, Wisconsin, USA). Sanger sequencing was performed using an ABI PRISM 3730XL Genetic Analyzer and BigDye Terminator Cycle Sequencing Kit v. 3.1 (Life Technologies, New York, USA), with results analyzed on Chromas LITE v 2.01 software (Technelysium, South Brisbane, Australia).

Examination of CATIE findings to assess consistencies of findings

NCBI accession (rs) numbers for 492,364 SNPs and p-values of treatment response associations from a previous GWAS study of treatment response in the CATIE study were examined.[1] There were differences in the study design and chronicity of patients enrolled in CATIE as compared to our first episode sample, and this is a consideration in the direct comparison of results across studies. However, noting the importance of examining findings in additional datasets, assessing the consistency of genetic patterns associated with response using that data may be helpful for many reasons. The primary microarray platforms used for both were from Affymetrix, increasing the likelihood of overlap of directly assayed SNPs in each. Second, the previously published GWAS of treatment response[1] stratified treatments, which allowed for the specific assessment of risperidone treated subjects, which was the primary treatment focus of our study population as well. The BPRS was our primary outcome variable of interest, and these items also make up a significant component of the PANSS which was the primary outcome variable in CATIE. Additionally, the efforts to examine time to treatment effects in CATIE found that using a factor assuming a 30 day exposure to drug is needed to assess response. This time frame is similar to the 6 week treatment period in our study. Finally issues of ethnic heterogeneity were handled in a similar fashion for both studies.

To assess direct comparisons of SNP findings across studies, we queried SNPs in CATIE to determine the level of significance for variants directly assessed in both studies. We also more broadly examined other SNPs in the primary genes of interest from our study (*GRM5*,

GRM7, *GRID2*) to examine patterns of nominal significance in each study and whether there were commonalities in these findings. Pairwise LD patterns for this effort were examined using the SNP Annotation and Proxy Search (SNAP) software using 1000 Genomes data as a reference.[2] This approach limits LD assessments to variants within 500kb of one another.

Pathway and functional annotation analysis of SNPs related to treatment response

Data were analyzed through the use of Qiagen's Ingenuity Pathway Analysis (IPA, Redwood City, CA http://www.qiagen.com/ingenuity). This software is used to analyze data derived from SNP microarrays to better understand the relationships of phenotypic outcome variables with larger biological or chemical systems using a structured repository of approximately 5 million individual findings describing relationships between molecules, diseases, and biological functions. Using the top 500 associated SNPs from each dataset, we took two approaches to more broadly assessing our findings and consistencies across study samples. The first tested the hypothesis that the top 500 SNPs from each study were over-represented in genes related to glutamate signaling and the second was a hypothesis free analysis to see whether there were other relevant pathways or gene networks of interest overrepresented in top findings. An IPA core analysis was used to examine direct and indirect relationships between mapped targets from SNPs and the IPA gene and endogenous chemicals database. To test the glutamate-specific pathway hypothesis we created a custom list of glutamate genes generated from KEGG and gene ontology databases. Discovery analyses utilized all databases and related canonical pathways from IPA. P-values for gene networks or functional categories represent the degree of over-representation of our findings relative to that expected by chance. Fishers Exact p-values are reported for the glutamate network analysis and Benjamini-Hochberg (adjusted for multiple comparisons) p-values are reported for the discovery analysis.

References:

1. McClay JL, Adkins DE, Aberg K, Stroup S, Perkins DO, Vladimirov VI*, et al*. Genome-wide pharmacogenomic analysis of response to treatment with antipsychotics. *Mol Psychiatry* 2011; **16**(1)**:** 76-85.

2. Johnson AD, Handsaker RE, Pulit SL, Nizzari MM, O'Donnell CJ, de Bakker PI. SNAP: a web-based tool for identification and annotation of proxy SNPs using HapMap. *Bioinformatics* 2008; **24**(24)**:** 2938-2939.
