# Supplementary material for: Antipsychotic pharmacogenomics in first episode psychosis: a role for glutamate genes
Source: Transl Psychiatry. 2016 Feb 23;6(2):e739–. doi: 10.1038/tp.2016.10 (PMC4872428; doi:10.1038/tp.2016.10)
Supplement: Supplementary Table 1 [file tp201610x2.pdf]

Supplementary Table 1. Strongest associations from candidate gene study. EMP1: pointwise p-value, EMP2: experiment-wise p-value; * SNP is within window of +/- 50 kilobases from candidate gene *HOMER2*.

rs#	Gene	EMP1	EMP2
rs2069062	GRM7	0.00012	0.01157
rs1532544	*GRM7*	0.0004	0.03949
rs3804850	GRM7	0.00126	0.0979
rs598134	*GRM5*	0.00135	0.114
rs1472476	GRM7	0.00151	0.1227
rs17376808	*PLCB1*	0.00203	0.1528
rs332938	GRM7	0.00231	0.1772
rs12905235	*WHAMM**	0.00255	0.1879
rs10899699	*SHANK2*	0.00297	0.2174
rs11018463	*GRM5*	0.00311	0.2261
