# Supplementary material for: Antipsychotic pharmacogenomics in first episode psychosis: a role for glutamate genes
Source: Transl Psychiatry. 2016 Feb 23;6(2):e739–. doi: 10.1038/tp.2016.10 (PMC4872428; doi:10.1038/tp.2016.10)
Supplement: Supplementary Table 2 [file tp201610x3.pdf]

**Supplementary Table 2. Strongest associations from candidate gene study in schizophrenia-spectrum illness subset.** EMP1: pointwise p-value, EMP2: experiment-wise p-value; * SNP is within window of +/- 50 kilobases from candidate gene *SLC1A1*.

| rs# | Gene | EMP1 | EMP2 |
|---|---|---|---|
| rs2069062 | *GRM7* | 0.00019 | 0.02619 |
| rs598134 | *GRM5* | 0.0005 | 0.04511 |
| rs12273644 | *SLC1A2* | 0.0017 | 0.1411 |
| rs17752444 | *GRM7* | 0.00187 | 0.1465 |
| rs17096210 | *GRIA1* | 0.00264 | 0.1913 |
| rs516273 | *GRIK3* | 0.00296 | 0.2215 |
| rs1532544 | *GRM7* | 0.00275 | 0.2322 |
| rs3804850 | *GRM7* | 0.00402 | 0.2672 |
| rs653216 | *SHANK2* | 0.0038 | 0.276 |
| rs10974671 | *SPATA6L** | 0.00352 | 0.2832 |
