# Supplementary material for: Antipsychotic pharmacogenomics in first episode psychosis: a role for glutamate genes
Source: Transl Psychiatry. 2016 Feb 23;6(2):e739–. doi: 10.1038/tp.2016.10 (PMC4872428; doi:10.1038/tp.2016.10)
Supplement: Supplementary Table 3 [file tp201610x4.pdf]

Supplementary Table 3. 20 strongest associations with change on positive subscale of BPRS in genome-wide analysis.

Rank	Chromosome	rsID	Gene	Region	$-\log_{10}$ p-value
1	8	rs1561296	*CSMD1*	Intron	6.79
2	18	rs8091220	intergenic	-	6.60
3	18	rs4939668	intergenic	-	5.96
4	8	rs10504371	intergenic	-	5.77
5	8	rs6981424	intergenic	-	5.63
6	18	rs9957078	intergenic	-	5.48
7	2	rs1561296	*SPATS2L*	Intron	5.37
8	13	rs1330968	intergenic	-	5.11
9	4	rs2071680	*ZFYVE28*	Exon	5.09
10	1	rs10802563	*ACTN2*	Downstream	5.02
11	3	rs2367607	intergenic	-	4.97
12	5	rs4398676	*LPCAT1*	Intron	4.96
13	8	rs2624095	*CSMD1*	Intron	4.82
14	20	rs6046528	*RIN2*	Downstream	4.79
15	9	rs773146	*NMRK1*	Downstream	4.77
16	20	rs6017147	*MYBL2*	Intron	4.58
17	1	rs2076590	*FAM212B*	Downstream	4.56
18	14	rs1956243	intergenic	-	4.50
19	18	rs12954691	*MRO*	Upstream	4.50
20	5	rs1191746	intergenic	-	4.46
