# Supplementary material for: Antipsychotic pharmacogenomics in first episode psychosis: a role for glutamate genes
Source: Transl Psychiatry. 2016 Feb 23;6(2):e739–. doi: 10.1038/tp.2016.10 (PMC4872428; doi:10.1038/tp.2016.10)
Supplement: Supplementary Table 4 [file tp201610x5.pdf]

Supplementary Table 4. 20 strongest associations with change on negative subscale of BPRS in genome-wide analysis.

Rank	Chromosome	rsID	Gene	Region	$-\log_{10}$ p-value
1	5	rs13172193	intergenic	-	4.95
2	2	rs2368445	intergenic	-	4.83
3	12	rs11107334	intergenic	-	4.81
4	1	rs17033865	intergenic	-	4.80
5	3	rs11915963	*CACNA1D*	Intron	4.67
6	12	rs11108366	*HAL*	Intron	4.65
7	1	rs10489747	*RXRG*	Intron	4.64
8	2	rs6743618	*PKP4*	Intron	4.63
9	11	rs10837340	*OR52J1P*	Upstream	4.61
10	16	rs3764286	*USP10*	Intron	4.55
11	11	rs1466267	*OR52E3P*	Downstream	4.50
12	10	rs2994684	intergenic	-	4.50
13	3	rs879716	*CLSTN2*	Intron	4.47
14	21	rs2823794	intergenic	-	4.45
15	10	rs4550135	intergenic	-	4.44
16	11	rs17538527	*OR9Q1*	Intron	4.37
17	8	rs272610	intergenic	-	4.35
18	10	rs7907704	intergenic	-	4.33
19	1	rs3120133	*ABCA4*	Intron	4.31
20	1	rs3120133	*ABCA4*	Intron	4.31
