# Supplementary material for: Antipsychotic pharmacogenomics in first episode psychosis: a role for glutamate genes
Source: Transl Psychiatry. 2016 Feb 23;6(2):e739–. doi: 10.1038/tp.2016.10 (PMC4872428; doi:10.1038/tp.2016.10)
Supplement: Supplementary Table 5 [file tp201610x6.pdf]

Supplementary Table 5. Stratified analysis of *GRID2* rs1875705. BPD: Bipolar disorder, MDD: major depressive disorder, CPZ: chlorpromazine equivalents.

	White (n=37)		Black (n=35)		Hispanic/Asian/ Native American (n=14)	
Variable	β	p	β	p	β	p
BPD diagnosis	3.4	0.41	8.7	0.004	5.2	0.14
MDD diagnosis	7.6	0.03	-2.2	0.62	10.4	0.02
Baseline BPRS	0.4	0.001	0.54	<0.001	0.29	0.07
ln CPZ	-1.4	0.31	-1.8	0.17	-1.2	0.50
GRID2 rs1875705	-5.5	<0.001	-6.8	<0.001	-5.9	0.009
Model r^2	0.60		0.59		0.68	
