# Supplementary material for: Antipsychotic pharmacogenomics in first episode psychosis: a role for glutamate genes
Source: Transl Psychiatry. 2016 Feb 23;6(2):e739–. doi: 10.1038/tp.2016.10 (PMC4872428; doi:10.1038/tp.2016.10)
Supplement: Supplementary Table 6 [file tp201610x7.pdf]

Supplementary Table 6. 20 strongest associations from genome-wide analysis of schizophrenia-spectrum illness subset.

Rank	Chromosome	rsID	Gene	Region	$-\log_{10}$ p-value
1	8	rs687279	intergenic	-	7.84
2	4	rs9307122	*GRID2*	intron	7.65
3	4	rs1875705	*GRID2*	intron	7.65
4	4	rs994011	*GRID2*	intron	5.78
5	18	rs12954691	*MRO*	upstream	5.77
6	8	rs673745	intergenic	-	5.24
7	8	rs418269	intergenic	-	5.04
8	4	rs10939542	intergenic	-	5.03
9	8	rs804280	*GATA4*	intron	4.86
10	16	rs11864396	*RBFOX1*	intron	4.76
11	5	rs6594905	intergenic	-	4.69
12	7	rs16100	*NPY*	downstream	4.66
13	7	rs16101	*NPY*	downstream	4.66
14	6	rs12215657	*LAMA2*	intron	4.65
15	16	rs7499973	*WWOX*	intron	4.54
16	2	rs6746182	*LRPPRC*	upstream	4.54
17	21	rs3119486	intergenic	-	4.54
18	10	rs10886048	intergenic	-	4.50
19	1	rs10922670	*LRRC8B*	downstream	4.49
20	14	rs12590805	intergenic	-	4.49
