# Supplementary material for: Antipsychotic pharmacogenomics in first episode psychosis: a role for glutamate genes
Source: Transl Psychiatry. 2016 Feb 23;6(2):e739–. doi: 10.1038/tp.2016.10 (PMC4872428; doi:10.1038/tp.2016.10)
Supplement: Supplementary Table 7 [file tp201610x8.pdf]

**Supplementary Table 7. Functional annotation categories of genes containing SNPs highly associated with antipsychotic treatment response**

| Study Sample | Functions Annotation | Fishers Exact p-value (unadj) | BH p-value (adj) | Genes with evidence of involvement in neuritogenesis and/or neuronal development |
|---|---|---|---|---|
| First Episode | neuritogenesis | 2.30E-05 | 1.67E-03 | *ATF6, ATP8A2, CNTNAP2, DDAH1, EPB41L3, GRID2, LAMA1, MAP2, MYO5B, NUMB, PALLD, PIP5K1B, PPP3CA, PRKCE, RNF165, THBS4, UNC13A, WWOX* |
| | development of neurons | 8.42E-05 | 5.66E-03 | |
| | morphogenesis of neurites | 1.74E-03 | 5.17E-02 | |
| | | | | |
| CATIE Risperidone | development of neurons | 2.72E-09 | 6.80E-07 | *CAST, CTNNA2, CUX2, DMD, DSCAM, FGFR2, ITPR1, LRRC4C, MPZ, MYO16, NRCAM, NRG1, NRP1, NTF3, NYAP2, PRKG1, PTPRE, RIMS1, ROBO2, ROR1, RYR2, SHANK2, TIAM2, TRIM9, TRPC1, WWOX* |
| | neuritogenesis | 3.53E-09 | 7.79E-07 | |
| | morphogenesis of neurites | 5.57E-08 | 6.95E-08 | |
| | | | | |

BH: Benjamini-Hochberg adjustment for multiple comparisons
