# Supplementary figures and images for: Antipsychotic pharmacogenomics in first episode psychosis: a role for glutamate genes
Source: Transl Psychiatry. 2016 Feb 23;6(2):e739–. doi: 10.1038/tp.2016.10 (PMC4872428; doi:10.1038/tp.2016.10)

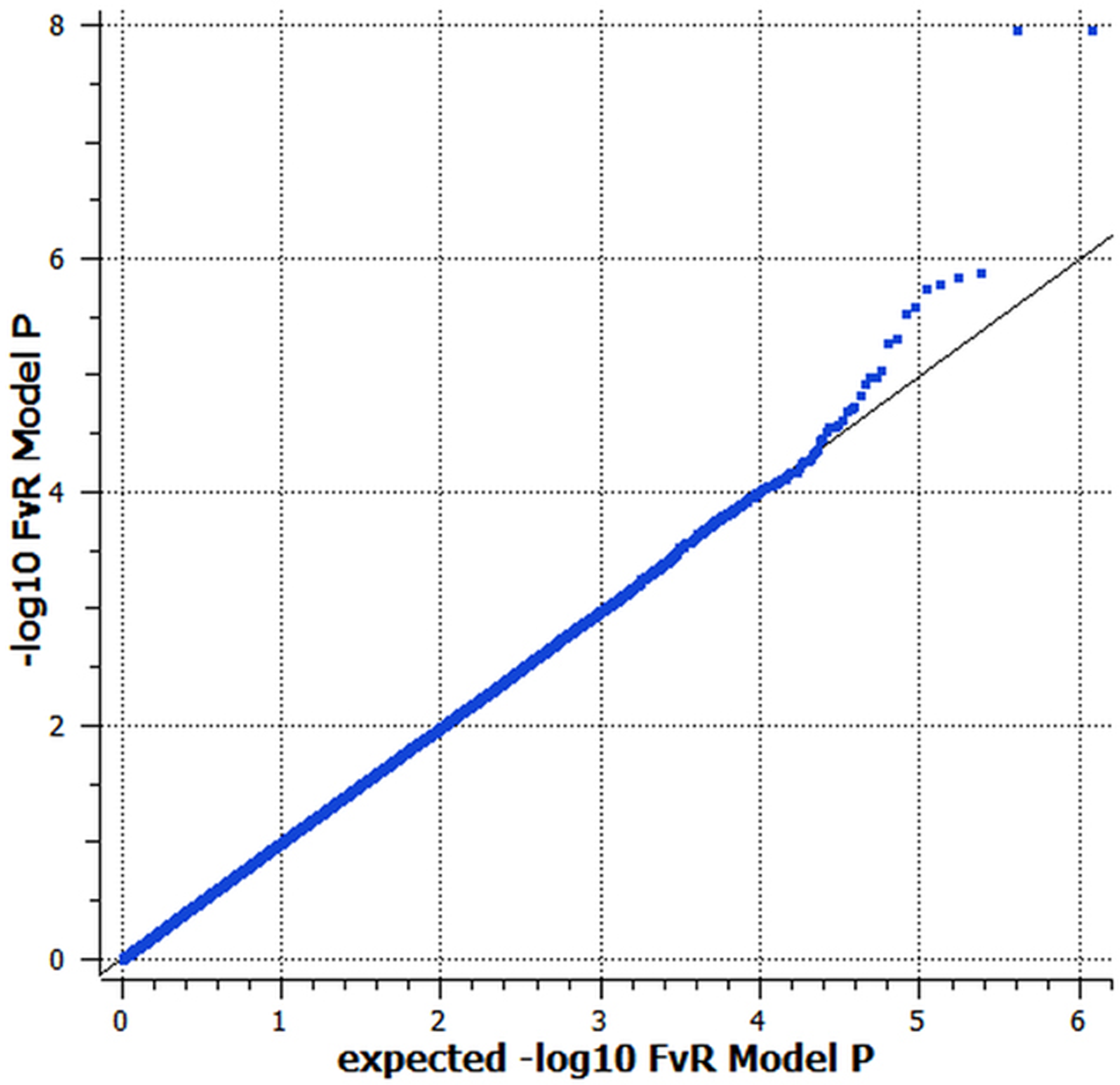

Supplement: Supplementary Figure 1 [file tp201610x9.tif]

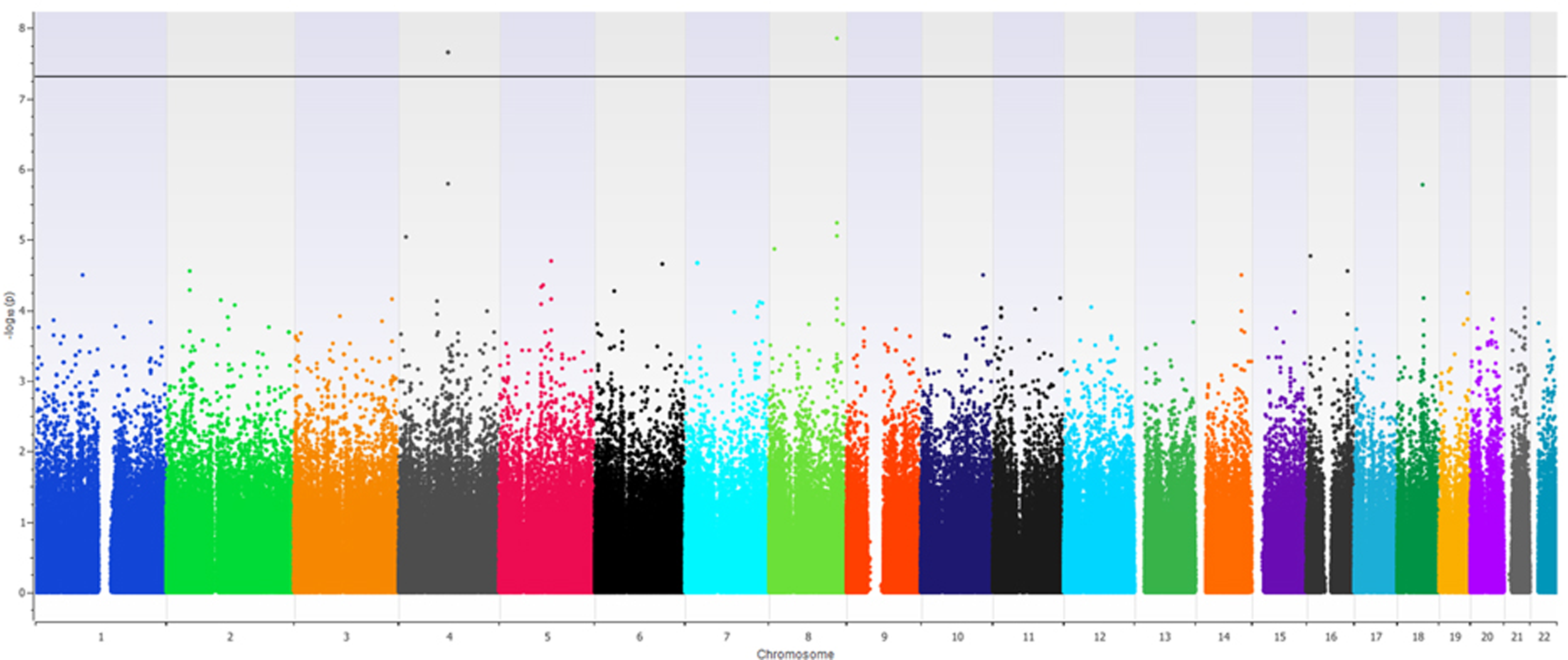

Supplement: Supplementary Figure 2 [file tp201610x10.tif]

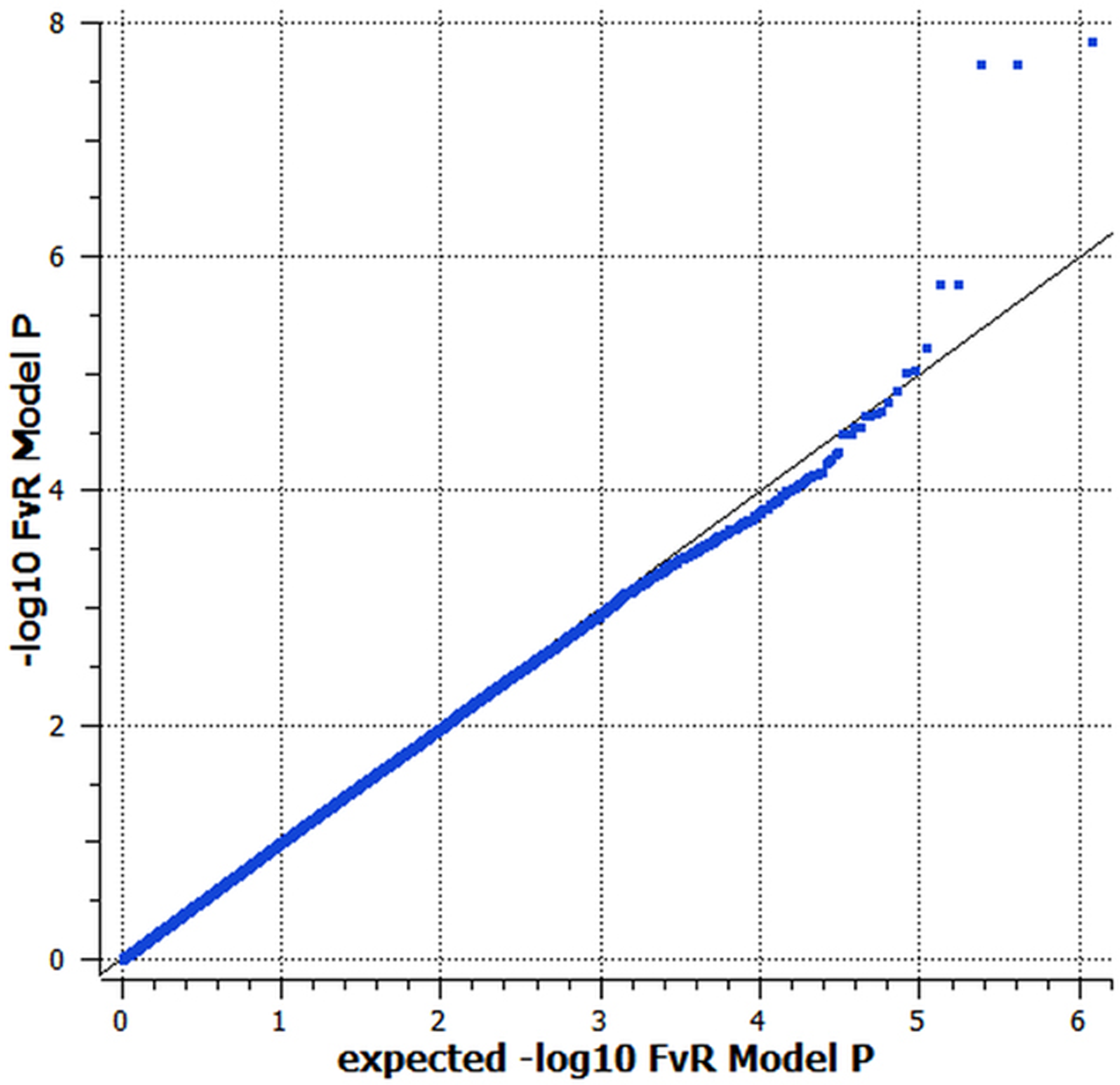

Supplement: Supplementary Figure 3 [file tp201610x11.tif]
